# Ultrasonographic Evaluation of Parathyroid Hyperplasia in Dialysis Patients

**DOI:** 10.1100/tsw.2006.273

**Published:** 2006-12-15

**Authors:** Drasko Pavlovic, Hrvojka Tomic Brzac

**Affiliations:** ^1^ Sestre Milosrdnice University Hospital, Vinogradska c.29, 10000 Zagreb, Croatia; ^2^ University Hospital Zagreb, Kispaticea 12, 10000 Zagreb, Croatia

**Keywords:** secondary hyperparathyroidism, parathyroid gland hyperplasia, ultrasonography

## Abstract

Secondary hyperparathyroidism (SHPT) is one of the most common complications in patients with chronic kidney disease (CKD). Bone and mineral disorders, increased morbidity and mortality are the consequences of SHPT. Therefore, prevention and control of hyperparathyroidism is one of the main objectives in the management of patients with CKD, particularly in dialysis patients.It is well known that SHPT in CKD is not only a state of increased parathyroid hormone (PTH) synthesis and secretion, but more importantly, it is a state of parathyroid gland (PTG) hyperplasia. The serum concentration of intact PTH is the main method used to assess PTG overactivity. Unfortunately, estimating the size and shape of the PTG in SHPT diagnosis, i.e., parathyroid hyperplasia, is still neglected. Among the various procedures, ultrasonography could be the method of choice to detect PTG size and shape because of its simplicity and noninvasiveness. This method can be sufficiently sensitive to distinguish diffuse and nodular hyperplasia.

## INTRODUCTION

Secondary hyperparathyroidism (SHPT) is one of the most common and most serious complications among patients with chronic kidney disease (CKD). Bone disease is a well-known consequence of SHPT, but today there is increasing evidence that SHPT is associated with increased morbidity and mortality of patients with CKD, particularly in stage 5[1]. Therefore, prevention and control of hyperparathyroidism is one of the main objectives in the management of CKD patients, particularly dialysis patients. Serum concentration of intact parathyroid hormone (PTH) currently is the main method used to assess parathyroid gland (PTG) overactivity. Unfortunately, estimating the size and shape of the PTG in SHPT diagnosis, i.e., parathyroid hyperplasia, is still neglected.

Despite the appearance of new drugs, i.e., new phosphate binders, vitamin D analogs, and calcimimetics, prevention and treatment of SHPT often is still unsuccessful. Underestimation of the importance of PTG hyperplasia can be a major factor for failed medical treatment of SHPT.

## PATHOGENESIS OF PARATHYROID TISSUE HYPERPLASIA IN CKD

Although a correlation between PTG weight and the severity of hyperparathyroidism was observed 40 years ago, the pathogenesis of abnormal PTH synthesis, secretion, and action among CKD patients received much more attention in the past than the pathogenesis of parathyroid hyperplasia.

As with other endocrine organs, PTG overactivity is associated with hypertrophy (increased cell volume) and hyperplasia (increased cell number)[2]. The characteristic of PTG hyperplasia in CKD patients is initially diffuse and polyclonal, after which nodular changes, i.e., monoclonal hyperplasia, occur.

The most important factors involved in the stimulation of PTH synthesis and secretion are insufficient production of calcitriol, phosphate retention, and calcium deficiency[3]. All three factors are also involved in PTG hyperplasia.

*In vitro* and *in vivo* studies have shown that calcitriol, which acts on a specific vitamin D receptor (VDR), negatively regulates pre-pro PTH gene transcription and suppresses PTH synthesis[4]. Calcitriol also controls parathyroid cell proliferation. Its effect on target cells depends on the content of a specific VDR. There is a significant reduction of VDR content in parathyroid cells of patients with CKD, particularly in areas of nodular hyperplasia. Since VDR expression is up-regulated by calcitriol, an insufficient level of calcitriol in renal insufficiency contributes to a reduction of VDR number. Therefore, a reduced level of calcitriol, a reduced number of VDR, and also postreceptor defects of vitamin D action contribute not only to PTG overactivity, but also to PTG hyperplasia[3,4].

The most important regulator of PTH secretion is extracellular calcium concentration. It acts through a specific calcium-sensing receptor (CaR)[5]. *In vivo* and *in vitro* studies have shown reductions of CaR in hyperplastic PTGs. The decrease is more marked in nodular hyperplasia, just as in VDR. In SHPT, a sigmoidal curve, which best shows the relationship between PTH secretion and extracellular calcium level, is shifted to the right and upwards, i.e., more calcium is needed to suppress PTH secretion. The presence of nodules in hyperplastic glands with low levels of CaR may explain these changes in the sigmoidal curve[5,6].

Phosphate retention contributes to SHPT indirectly by decreasing calcitriol synthesis and inducing hypocalcemia. In addition, there is evidence that hyperphosphatemia has a direct effect in increasing PTH secretion. This effect is independent of extracellular calcium concentration[7]. The role of the recently discovered phosphaturic factor fibroblast growth factor 23 (FGF 23) in pathogenesis is not yet completely understood. It is known that the level is increased in CKD. FGF 23 suppresses active vitamin D production in the kidney and may predict future development of refractory hyperparathyroidism in dialysis patients[8].

The recent work of Dusso et al.[9] sheds new light on the pathogenesis of parathyroid hyperplasia. The enhanced parathyroid expression of transforming growth factor alpha (TGF-$\alpha$), a very potent growth promoter and its receptor, edpidermal growth factor receptor (EGFR), probably have the most significant role in parathyroid hyperplasia. Even more, the reduction of the VDR in hyperplastic PTGs is mediated by activation of EGFR by TGF-$\alpha$[9].

From the clinical point of view, hyperplastic PTGs are much larger, PTH level is much higher, there is significant reduction of VDR and CaR number (particularly in nodular hyperplasia), there is increased basal secretion of PTH, and there is a shift of calcium/PTH curve upward. The result is that medical treatment of such patients is very often unsatisfactory[2,10].

## PARATHYROID SONOGRAPHY

Various noninvasive methods, technetium/thallium scintigraphy, computed tomography, magnetic resonance, and ultrasonography have been used for the identification and localization of PTGs. Each method has its advantages and disadvantages[11]. Ultrasonography is a simple, cheap, and noninvasive method. In the hands of an experienced operator, ultrasonography has sufficient specificity to assess not only localization, but also the size, shape, and type of PTG hyperplasia.

We began to use ultrasound in the diagnosis and localization of the PTG about 30 years ago. At that time, we used a device of 3.5 MHz with the basic goal of localizing an enlarged PTG (Fig. 1). We believed that it was important for the surgeon to localize the PTG before surgery, and to enable him to plan the procedure and carry it out as safely and quickly as possible[12].

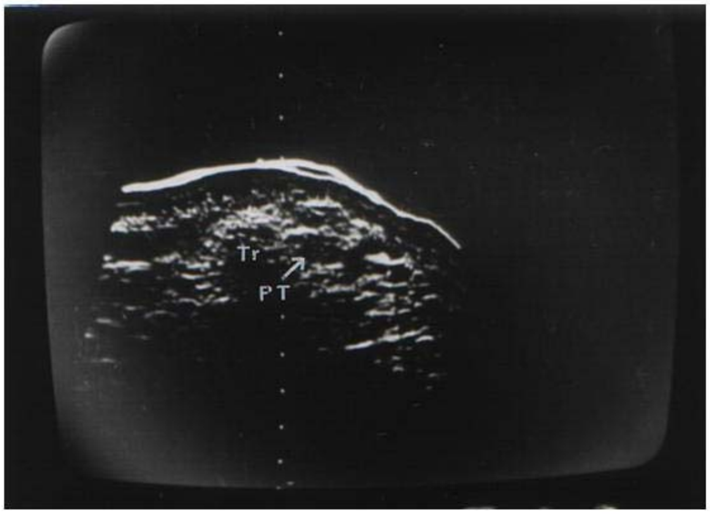

**FIGURE 1.** Echographic presentation of PTG with compound ultrasonography and 5-MHz probe.

Since that time, our understanding of the pathogenesis of SHPT, and especially of hyperplasia in PTGs, has advanced significantly. Numerous studies have shown that hyperplasia, or the disrupted growth of the PTG, is the most significant in CKD[2,13,14,15]. Japanese clinicians**,** based on their experience with many parathyroidectomies and modern ultrasound equipment, have shown that CKD patients exhibit signs of hyperplasia very early in CKD, first diffuse and later nodular hyperplasia[6,15] (Fig. 2). They have also shown that secondary hyperthyroidism can be characterized by asymmetrical enlargement of the PTG, i.e., a patient may have PTGs that are smaller with diffuse hyperplasia and larger ones with nodular hyperplasia at the same time[15].

## PATIENTS AND METHODS

We examined 120 patients, 63 males and 57 females, mean age 45 years (14–79), who have been dialyzed for an average of 7.8 (0.5–22) years. We performed parathyroid sonography using 2-D high-resolution sonography with a 10-MHz linear probe for small parts and color Doppler. PTG volume was calculated according to the formula $V = (a \times b \times c) \times \Pi/6$. Intact PTH was measured by immunoradiometric assay (normal range 9–62 pg/ml).

## RESULTS

In 120 patients, 362 enlarged PTGs were found (an average of three per patient).

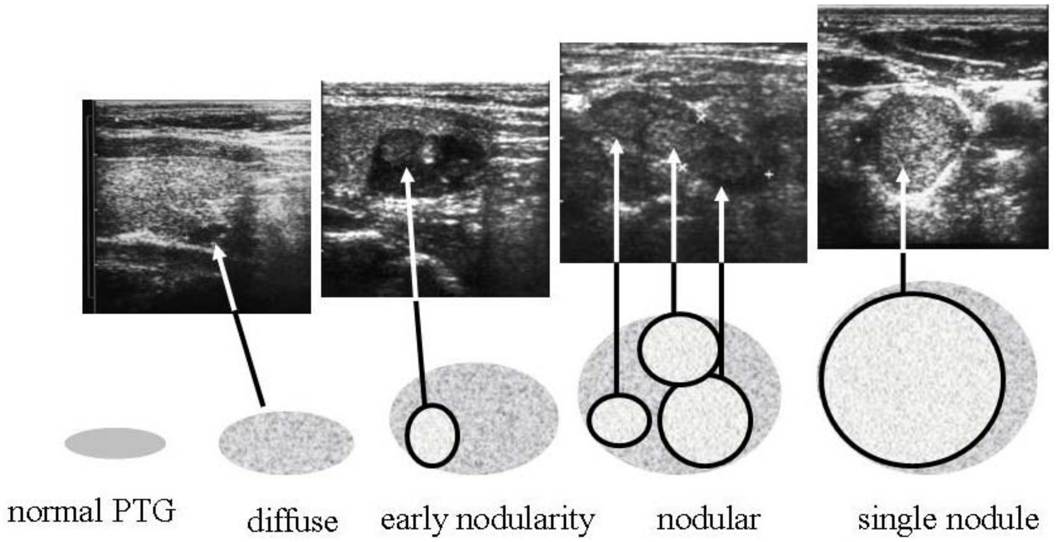

**FIGURE 2**. Progression of renal hyperparathyroidism. Normal PTGs are not visible with ultrasound. Hyperplastic glands with diffuse hyperplasia are hypoechogenic, with homogeneous echoes. Early nodularity is represented with a small isoechogenic nodule within the enlarged hypoechogenic gland. The next step is multiple nodules in the PTG. The end step of development of nodularity is one nodule that takes over the whole gland.

In 58 (48%) patients, uniform echoes, i.e., diffuse hyperplasia of the PTG, were found; 34 patients (28%) had a nodular pattern of the PTG; and in 28 patients (23%), we detected degenerative changes of the PTG. By use of color Doppler sonography, paranodular vascularity was found only in PTGs with nodular hyperplasia (38%), while in diffuse PTG hyperplasia, hypervascularity was found in 22%, internal vascularity in 28%, and no vascularity in 12% of PTGs. We observed the higher PTH level in patients with nodular hyperplasia and paranodular vascularity of the PTG, i.e., PTH was 1212.3 pg/ml, than in patients with diffuse hyperplasia and internal vascularity (PTH 765.1 pg/ml) or hypervascular capsule (PTH 961.3 pg/ml) ($p < 0.01$ ) (Table 1).

**TABLE 1**
**PTH Level Depending on Ultrasonographic Finding**

| 2-D sonography | Color Doppler | PTH (pg/ml) |
|---|---|---|
| Diffuse hyperplasia | internal vascularity | 765,1 |
| | hypervascular capsule | 961,3 |
| Nodular hyperplasia | paranodular vascularity | 1212,3* |
| p < 0.01 | | |

## DISCUSSION

Using modern ultrasound equipment, we have been able to observe enlarged PTGs (Fig. 3), then to measure all three dimensions, and calculate the volume with a satisfactory level of accuracy. Hyperechogenic echoes,

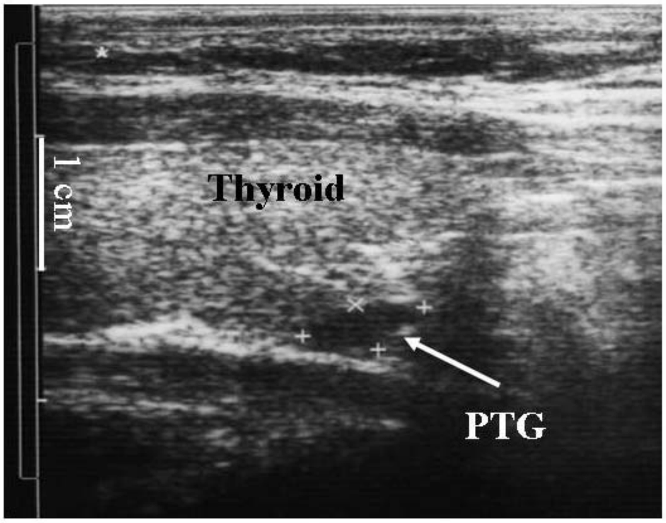

**FIGURE 3**. PTG with diffuse hyperplasia in 41-year-old woman, on dialysis treatment 5 years, PTH 129.2 pg/ml (longitudinal section, linear probe 10 MHz).

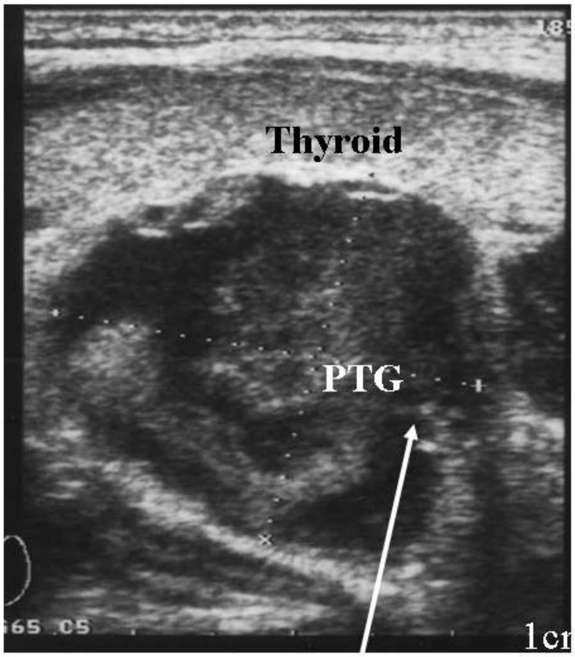

**FIGURE 4**. PTG with nodular changes in 43-year-old woman, on dialysis treatment 11 years, PTH > 1500 pg/ml (longitudinal section, linear probe 13 MHz). Hyperechogenic echoes that correspond to nodular changes.

which most probably indicate nodules in the PTG, often can be observed in enlarged PTGs (Fig. 4). Unfortunately, it is not possible to prove that this is an indication of nodular hyperplasia. Genetic analysis of a fine-needle aspiration sample might be of value, but it is not useful in routine work. Only postoperative histological analysis can establish the presence of nodular hyperplasia. However, based on the linkage between enlargement of PTGs and the type of hyperplasia, there is little doubt that in glands larger than 0.5 $cm^3$ or heavier than 0.5 g, and with hyperechogenic echoes within the gland, nodular hyperplasia probably exists[15]. The use of power-Doppler ultrasonography can contribute to the differentiation of diffuse from nodular hyperplasia. Onoda and others have shown that increased vascularity around or within the glands indicates nodular hyperplasia in 80% of cases. In glands with less vascularity, it is more frequently a case of diffuse hyperplasia[16]. We have also found similar results with very small differences in the criteria of vascularity[17] (Figs. 5, 6, 7, 8).

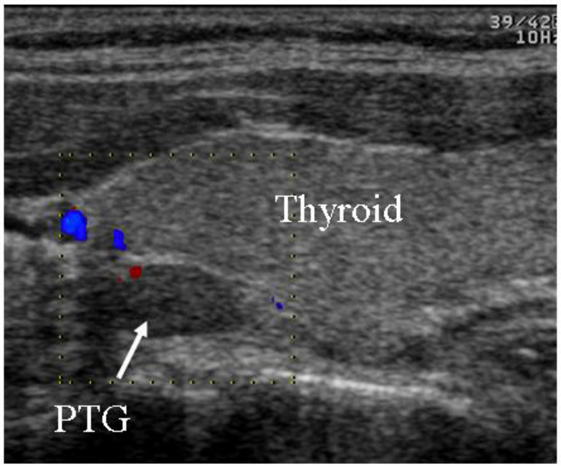

**FIGURE 5.** PTG without vascularity in 42-year-old woman, on dialysis treatment 5 years, PTH 166.1 pg/ml (longitudinal section, linear probe 13 MHz).

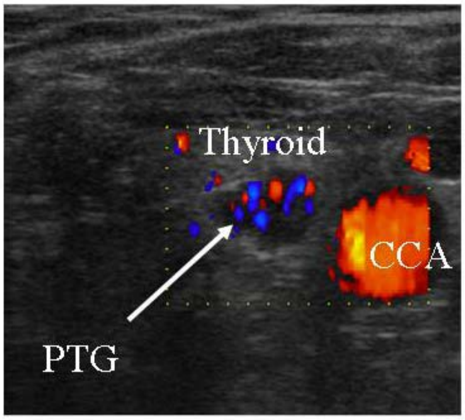

**FIGURE 6.** PTG with intensive internal vascularity in 52-year-old woman, on dialysis treatment 18 years, PTH 712.3 pg/ml (transversal section, probe 10 MHz).

However, it is not sufficient only to determine the size and type of PTG hyperplasia. Within the PTG, calcificates or cysts and degenerative changes can often be detected (Figs. 9, 10). In patients with such glands, PTH is significantly elevated, but it is lower in patients with clear nodular hyperplasia[17]. In addition, by means of ultrasound, it is possible sometimes to observe unusual changes in PTGs in CKD patients, i.e., inflammation and hemorrhagic changes[18,19].

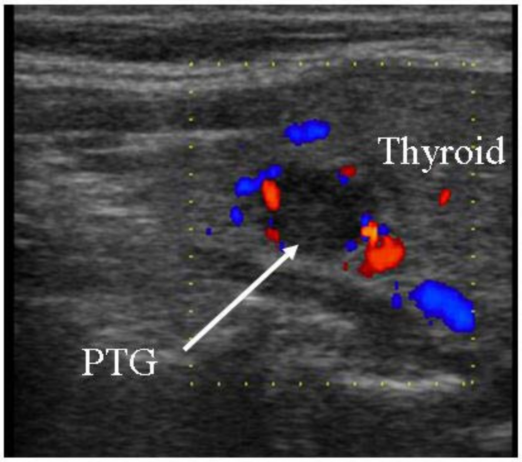

**FIGURE 7.** PTG with hypervascular capsule in 44-year-old woman, on dialysis treatment 3 years, PTH 912.3 pg/ml (longitudinal section, probe 10 MHz).

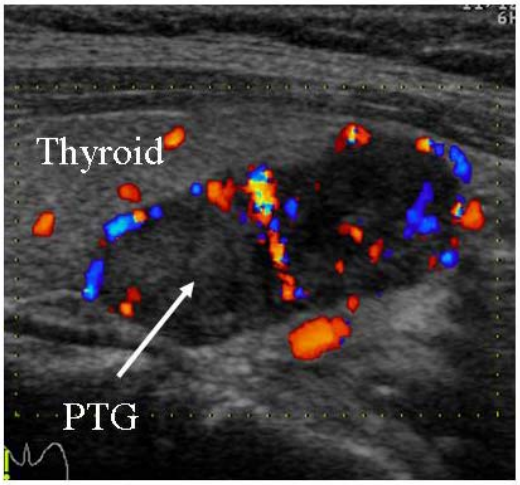

**FIGURE 8.** PTG with paranodular vascularity in 44-year-old woman, on dialysis treatment 4.5 years, PTH 1230.2 pg/ml (longitudinal section, probe 13 MHz).

What is the advantage of using ultrasound evaluation of PTGs? By means of sonography, it is possible to detect the size, shape, and position of PTGs. Furthermore, a different echo pattern of PTGs can be observed. Uniform echoes are more frequently found in smaller glands (diffuse hyperplasia). A nodular echo pattern and occasionally degenerative changes (calcificates, cystic and fibrotic changes) are found in larger PTGs (nodular hyperplasia). By use of color Doppler, a difference in vascularity can be detected[16,17].

The importance of determining the type of hyperplasia in the treatment of SHPT is because the reduced number of VDR and CaR in the glands will most probably predict resistance to medical treatment of SHPT[20], and parathyroidectomy could be recommended[21].

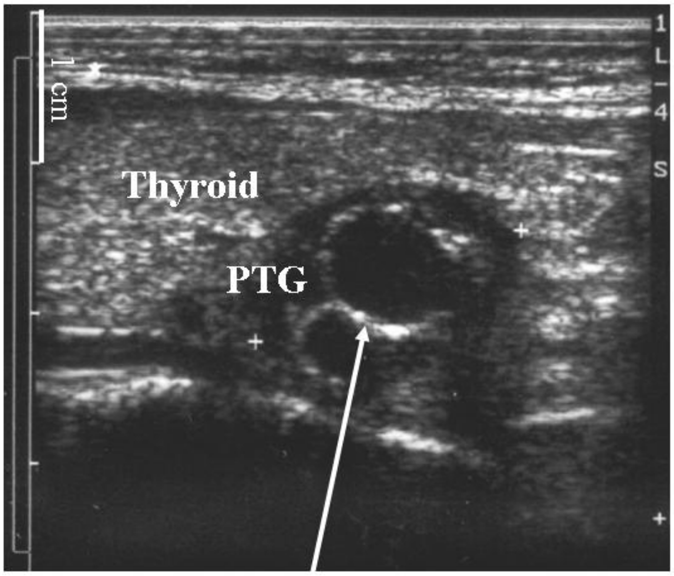

**FIGURE 9.** PTG with cystic degeneration in 50-year-old woman, on dialysis treatment 7 years, PTH 626.6 pg/ml (longitudinal section, linear probe 10 MHz).

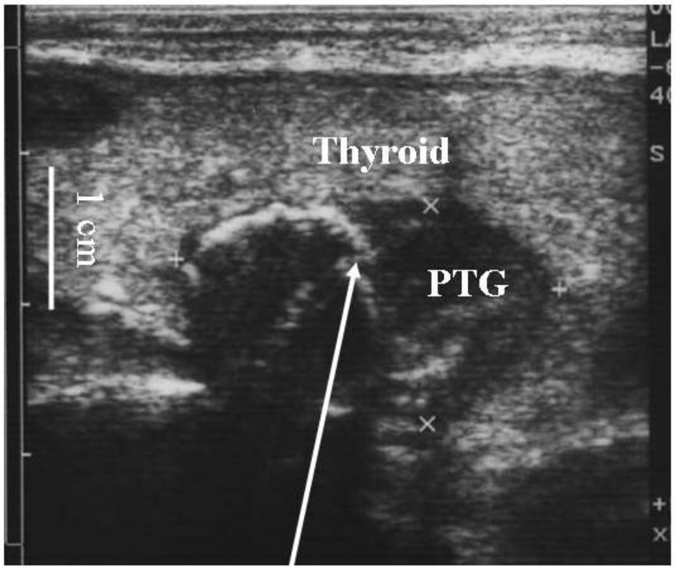

**FIGURE 10.** PTG with calcifications in 40-year-old woman, on dialysis treatment 12 years, PTH 1775.9 pg/ml (longitudinal section, linear probe 10 MHz).

Based on our experience to date, and on that of others (primarily Japanese authors), therapy can be prescribed on the basis of PTH level and ultrasound findings, i.e., PTG size. In patients with smaller PTGs, i.e., diffuse hyperplasia, treatment by vitamin D, analogs of vitamin D, or calcimimetics is recommended[22]. In patients with larger glands, i.e., nodular hyperplasia, treatments with the above-cited drugs most probably will not be effective[20,23]. In patients with one or two large PTGs, inactivation with ethanol can be used. More than 20 years ago, Solbiati and colleagues described a method for the inactivation of the PTG with ethanol[24]. This method was further developed in other countries, primarily in Japan[25]. Our experience has also shown that this method can be useful[26]. By the direct injection of vitamin D analog maxacalcitol, Japanese authors have shown not only a fall in the PTH level, but also apoptosis of parathyroid cells[27]. Unfortunately, this method has not found a broader application for now, and its long-term effectiveness has not been sufficiently proven. In patients with three or four glands with nodular changes and a high value of parathormon, a parathyroidectomy is recommended[21,25].

In conclusion, we wish to emphasize that we agree with the position of Parfitt[2] that hyperparathyroidism of chronic renal failure is a disorder of growth, and studying the pathogenesis and monitoring the effects of treatment size of PTGs is paramount and ultrasound could be the method of choice for that purpose[2]. As an inexpensive and noninvasive method, ultrasound can be extremely useful for this purpose, but an experienced ultrasound technician is required.
